# [Retracted] Silencing cyclin‑dependent kinase inhibitor 3 inhibits the migration of breast cancer cell lines

**DOI:** 10.3892/mmr.2023.13075

**Published:** 2023-08-22

**Authors:** Miao Deng, Jianguang Wang, Yanbin Chen, Like Zhang, Gangqiang Xie, Qipeng Liu, Ting Zhang, Pengfei Yuan, Dechun Liu

Mol Med Rep 14: 1523–1530, 2016; DOI: 10.3892/mmr.2016.5401

Following the publication of this paper, it was drawn to the Editor's attention by a concerned reader that the GAPDH control western blotting data shown in Fig. 1C were strikingly similar to data appearing in different form in another article written by different authors at different research institutes [Chen Y, Guo Y, Yang H, Shi G, Xu G, Shi J, Yin N and Chen D: TRIM66 overexpression contributes to osteosarcoma carcinogenesis and indicates poor survival outcome. Oncotarget 6: 23708–23719, 2015]. Moreover, a pair of data panels showing the results from cell-cycle experiments purportedly performed under different experimental conditions in Fig. 4A appeared to be strikingly similar.

Owing to the fact that the contentious data in the above article were already under consideration for publication prior to its submission to *Molecular Medicine Reports*, the Editor has decided that this paper should be retracted from the Journal. The authors were asked for an explanation to account for these concerns, but the Editorial Office did not receive a reply. The Editor apologizes to the readership for any inconvenience caused.

